# Role of Viscous Dissipative Processes on the Wetting of Textured Surfaces

**DOI:** 10.1038/srep14159

**Published:** 2015-09-22

**Authors:** H. S. Grewal, Hong Nam Kim, Il-Joo Cho, Eui-Sung Yoon

**Affiliations:** 1 Center for Biomicrosystems, Korea Institute of Science and Technology (KIST), Seoul, Republic of Korea

## Abstract

We investigate the role of viscous forces on the wetting of hydrophobic, semi-hydrophobic, and hydrophilic textured surfaces as second-order effects. We show that during the initial contact, the transition from inertia- to viscous-dominant regime occurs regardless of their surface topography and chemistry. Furthermore, we demonstrate the effect of viscosity on the apparent contact angle under quasi-static conditions by modulating the ratio of a water/glycerol mixture and show the effect of viscosity, especially on the semi-hydrophobic and hydrophobic textured substrates. The reason why the viscous force does not affect the apparent contact angle of the hydrophilic surface is explained based on the relationship between the disjoining pressure and surface chemistry. We further propose a wetting model that can predict the apparent contact angle of a liquid drop on a textured substrate by incorporating a viscous force component in the force balance equation. This model can predict apparent contact angles on semi-hydrophobic and hydrophobic textured surfaces exhibiting Wenzel state more accurately than the Wenzel model, indicating the importance of viscous forces in determining the apparent contact angle. The modified model can be applied for estimating the wetting properties of arbitrary engineered surfaces.

The theoretical estimation of wetting properties of engineered surfaces has long been of interest to researchers due to its broad range of applications such as self-cleaning and floatation, and in the reduction of hydrodynamic drag force. Conventional Wenzel^1^ and Cassie-Baxter^2^ wetting models or their derivatives essentially provide a first-order estimation of the wetting properties for textured surfaces. However, due to the presence of second-order effects^3^, theoretical estimations for geometrically complex surfaces demonstrate discrepancies with the experimental values. Moreover, many of the proposed models demonstrate limited capability in predicting the apparent or advancing contact angles of complex surfaces. Such discrepancies largely originate from the limited consideration of secondary processes, affecting the spreading dynamic of a droplet. Although wetting is a thermodynamic process controlled primarily via capillary force, the spreading of a liquid droplet is also controlled by secondary processes such as disjoining pressure, viscous force, and contact line friction^3^,^4^,^5^,^6^,^7^,^8^,^9^, effects believed to affect the wetting of an engineered surface.

Each secondary process operates dominantly at a specific scale (Fig. 1) and thus should be considered in response to the feature scale of a liquid droplet. At the microscale, long and short-range intermolecular forces dominate the spreading of a liquid film ahead of a droplet. The combined effect of these intermolecular forces influences the disjoining pressure and hence controls the extension of a precursor film^3^,^4^,^7^. At the mesoscale, the hydrodynamic rolling motion in the wedge section generates viscous forces contributing to the dissipation process^3^,^4^,^5^,^7^,^10^. The interaction between the secondary processes and the interfacial surface tension modulates local curvature and ultimately determines the apparent (advancing) contact angle^4^,^7^,^11^,^12^,^13^,^14^,^15^. The importance of local phenomena at the contact line in the determination of the contact angle is further supported by recent studies^16^,^17^,^18^,^19^,^20^,^21^, which indicate that the contact angle is mediated by the contact line rather than the contact area of a droplet (For further discussion on this subject, please see Erbil^22^). This observation indicates that the kinetics of a contact line dominantly controls the wetting.

Among the secondary processes, the role of a precursor film (disjoining pressure) on wetting has been explicitly explored for the last few years^7^,^11^,^12^. However, the number of investigations on the effects of viscous forces on the wetting of patterned surfaces is limited, as most of the studies exploring the effect of viscous forces were conducted on flat surfaces or under highly dynamic conditions^13^,^14^,^15^,^23^. Kavehpour *et al.*^14^ showed a direct influence of the capillary number (ratio of viscous force to surface tension)*, Ca* (2 × 10^−6^ to 3.2 × 10^−4^), on the advancing apparent contact angle (~10°) for flat surfaces. Rame and Garoff^15^ and Dussan *et al.*^24^ showed that for flat surfaces, the contact angle at the macroscale and mesoscale is influenced by *Ca*. In this study, the viscous forces showed significant change of the macroscopic apparent contact angle at *Ca* ≈ 0.1. Keller *et al.*^23^ reported the influence of viscosity on the apparent contact angle in a broad range of *Ca* values. Using phase field theory, Vedantam and Panchagnula^25^,^26^ developed a constitutive model for estimating the contact angle hysteresis and contact line friction of chemically heterogeneous surfaces. As the basic inputs, this model requires the advancing and receding contact angles, liquid-vapor interfacial energy, and the three-phase line tension. Considering that most engineered surfaces contain textures and that the motion of a droplet covers a broad range of dynamics, consideration of the effect of viscous force on textured surfaces is substantially needed. Previous studies have demonstrated that viscous dissipation plays a crucial role in the propagation of wetting front through the micro-pillar arrays^27^,^28^. The velocity of propagation was observed to be dependent upon the geometrical parameters of the pillars. Bayer and Megaridis^29^ also showed that the final value of a macroscopic contact angle is influenced by its dynamic history.

In this study, we investigate the role of viscous forces on various engineered surfaces and present a theoretical model that can predict apparent contact angles on arbitrary surfaces. For this purpose, first, we demonstrate the dynamics of a liquid droplet on contact with a surface using a high-speed camera. Then, the effects of viscosity are investigated by measuring the apparent contact angle of liquids with various viscosities (1–1270 mPa) on surfaces with different combinations of topography and surface chemistry. Based on the observations, we classify the conditions in which viscous forces play an important role. Finally, a model for predicting the apparent contact angle on an arbitrary engineered surface is presented by balancing the driving and resisting forces.

## Results an Discussion

### Dynamics of the initial spreading of a liquid droplet

When a droplet is gently placed on a solid surface (completely or partially wettable), it spreads rapidly until reaching equilibrium (Supplementary Movie S1). During this initial spreading, the advancing contact line generates an internal flow within a liquid drop. Here, the flow field inside of a drop can be classified into two domains^30^,^31^: (i) the flow in the bulk of a drop and (ii) the flow at the moving contact line (Supplementary Movie S2). The diverging flow field and eddies near the advancing contact line give rise to significant viscous forces^3^,^13^. These viscous forces are apparently different from those in the bulk, which have negligible effects on the apparent contact angle. The viscous force at the contact line interacts with the capillary force and controls the spreading rate during the quasi-static conditions.

**Figure 2:**
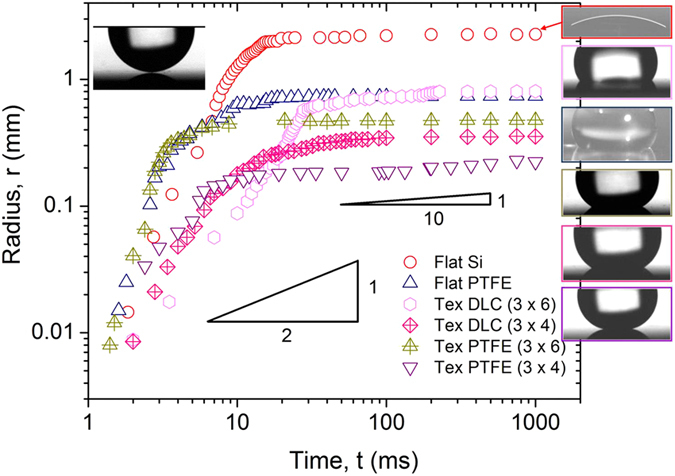
The initial spreading (*r* vs *t*) behaviour of the water droplet on flat and textured (tex) surfaces. The textured surfaces consisted of cylindrical pillars (3 μm high, 4 μm in diameter and 4 μm and 6 μm pitch. Inset shows the shape of a droplet for the corresponding surface at different times.

To demonstrate the effect of surface texture and chemistry on the dynamic motion of a liquid spreading, we observed the spreading dynamics using a high-speed camera and plotted the spreading radius as a function of time (Fig. 2). Although the spreading rate follows power-law, the values of exponents are different depending on the dominant resistive forces, showing a sharp transition at the ~10–20 ms time point. During the early spreading (before the transition point; ≤20 ms), the value of exponent *α* is ≥0.5, which results from the balance of capillary (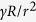) and inertial 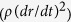 forces^32^. This scaling law was observed irrespective of the surface chemistry and texture. At time ≥20 ms, the spreading gradually transitions from an inertia-dominant to a viscous-controlled regime, during which the value of exponent α changed from 0.3 to 0.1 (Fig. 2). The spreading rate during this regime follows Tanner’s law^33^, which indicates viscous-dominated spreading.


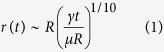


where *μ* is viscosity, *γ* is surface tension, *R* is the initial radius of a drop, *t* is time and *r* is the instantaneous radius of the contact line. During the viscous-dominated regime, the apparent contact angle of a moving contact line gradually attains a constant value under quasi-static conditions.

Furthermore, these results collectively indicate that surface chemistry and textures control the transition from the inertial to viscous-dominant regime and ultimately affect the spreading of a droplet. In this experiment, the inertia force was controlled identically (water droplet volume of 5 μl). Considering that the initial spreading is mediated by the force balance between capillary and inertia forces, the different spreading is mediated primarily by capillarity unless the viscosity changes.

### Role of viscous forces in wetting

In this section, the interaction between the viscous liquid and the engineered surfaces will be considered. We measured apparent contact angles with liquids with different viscosities (Supplementary Table S1) to demonstrate the effect of viscous forces on spreading and subsequently on the apparent contact angle. Liquids with various viscosities were prepared by mixing water and glycerol in different ratios. The viscosity increases as the content of glycerol increases, whereas the surface tension shows an opposite trend, although the change of surface tension is small compared to that of viscosity.

**Figure 3:**
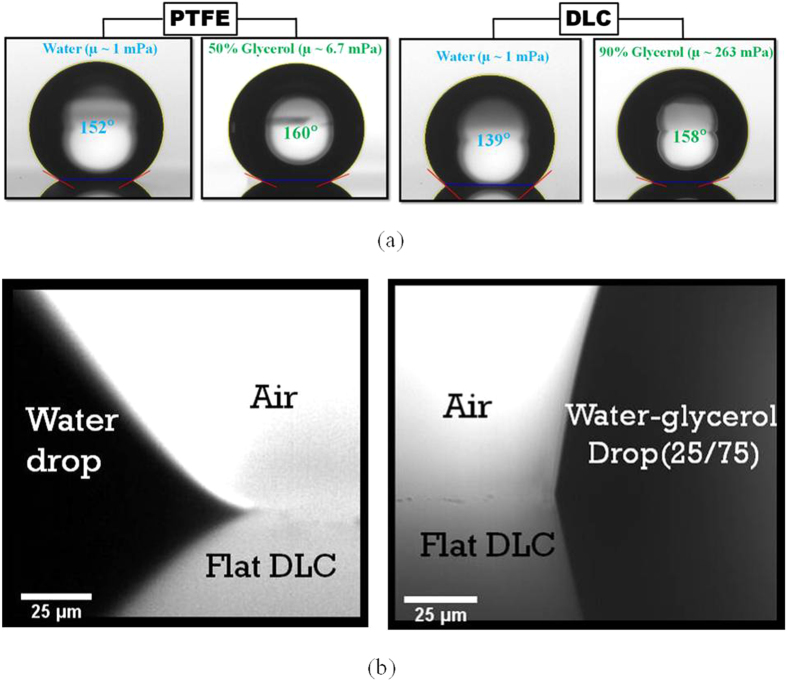
(**a**) Change in the macroscopic apparent contact angle of the droplet for textured surfaces with a change in liquid viscosity. (**b**) The effect of viscosity on the mesoscopic apparent contact angle of the droplet for a flat DLC surface. Droplets on the Si surface showed no effect of viscosity on the apparent contact angle at either length scale.

**Figure 4:**
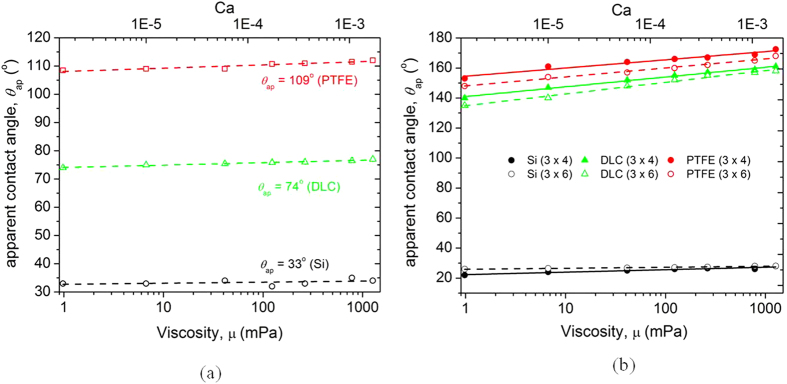
Effect of capillary number (viscosity) on the macroscopic apparent contact angle of the (**a**) flat and (**b**) textured surfaces.

The effect of viscosity was different depending on the surface textures (Figs 3 and 4). As shown in Fig. 4(a), the apparent contact angle of liquid droplets on flat substrates including Si, diamond-like coating (DLC) and polytetrafluoroethylene (PTFE) showed negligible change as viscosity increases (maximum ≈ 4° for 1000 times change of viscosity). The results for the flat surfaces are consistent with Rame and Garoff^15^, whereby they observed a difference of approximately 3.2° at *Ca* ≈ 0.1 (contact line velocity ≈ 3.2 × 10^−5^ m/s).

By contrast, the apparent contact angle was significantly affected by textured surfaces, especially for the hydrophobic (PTFE) and semi-hydrophobic (DLC) (surface with equilibrium contact angle slightly lower than 90°) surfaces (change of contact angle 20° and 23°, respectively). However, liquid droplets on hydrophilic textured surfaces (Si) showed nominal changes of apparent contact angle (change of contact angle by 2° to 6°). These results indicate that roughness enhances the dependence of contact angle on *Ca* (due to increase in viscous forces), as discussed by others^34^,^35^.

Considering the fact that the fluid flow in the viscous regime follows Stokes law, the viscous forces for the flat and textured (cylindrical pillars) surfaces scales as^36^,^37^


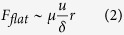



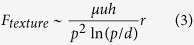


where *u* is the velocity, and *h*, *d* and *p* are the height, diameter and pitch of the pillars, respectively. Landau and Levich^38^ and Derjaguin^39^ estimated the thickness of the viscous boundary layer for a dynamic meniscus and found it is a function of *Ca*. Thus, for *Ca* ~ 1.6 × 10^−3^, the thickness of this boundary layer will be ~34 μm. The ratio of the viscous force for texture to that for the flat surface 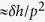
is equal to ~12 for *h* = 3 μm and ~40 for *h* = 10 μm. Evidently, the magnitude of the viscous force for a textured surface is much higher than that for a flat surface. From the experimental results in Fig. 3 and Fig. 4 and theoretical estimation, it can be concluded that the apparent contact angle of the textured surfaces increases with an increase in viscosity due to the amplification of viscous forces.

In case of flat surfaces, the absence of roughness gives rise to a relatively small change in viscous force, regardless of surface chemistry. In our observation, the effect of viscosity was only limited to the mesoscale and did not affect the macroscale contact angle. As an example, Fig. 3(b) shows the profile of droplets at the mesoscale for the DLC flat surface. Although the flat surfaces showed a marginal increase in the macroscopic apparent contact angle with an increase in viscosity, the mesoscopic contact angles were significantly different due to the viscous forces. However, the increase in viscous forces did not show any appreciable change for the Si flat surface and only a nominal change for the textured surfaces both at the macroscopic and the mesoscopic scales, which is due to the dominance of another dissipation mechanism (precursor film). Details will be described in the following section.

### The precursor film: an alternative dissipation mechanism in hydrophilic surfaces

To understand the insignificant effect of liquid viscosity on apparent contact angle for hydrophilic flat and textured surfaces, the difference in underlying energy dissipation mechanisms needs to be considered. In wetting process, dissipation mainly occurs through three different mechanisms: the precursor film (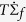), the viscous dissipation in the wedge (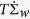) (mesoscale) and the line friction (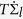)^3^.


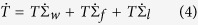


Precursor film is a result of attractive disjoining pressure, which promotes the spreading of a liquid film ahead of a droplet. Disjoining pressure is a combined effect of intermolecular forces: van der Waals (dispersion), electrostatic and structural (solvation, hydration)^12^,^40^. The disjoining pressure isotherm Π(*l*) is described as a derivative of the effective interface potential, ξ(*l*) with respect to *l* as^4^,^12^


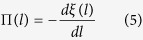


**Figure 5:**
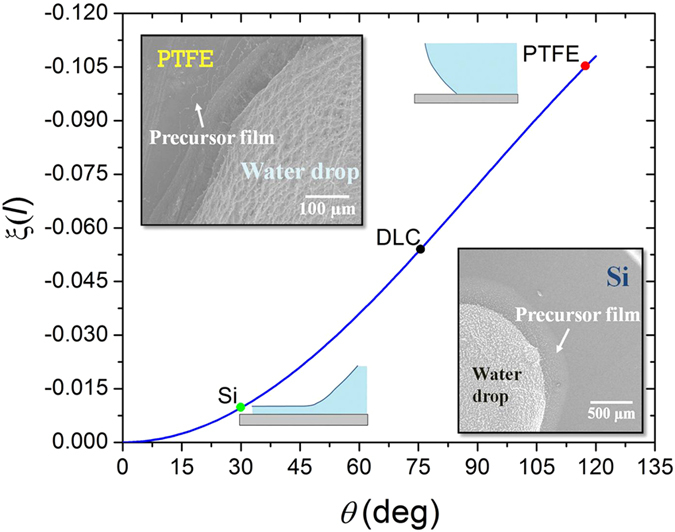
Relationship between effective interface potential, ξ(*l*) and the contact angle. The increase in ξ(*l*) indicates the increase in disjoining pressure (Eq. 4), which limits the extent of the precursor film. The frozen water droplet images shown in the inset for the flat Si and the PTFE surfaces complement this relation. The large extent of the precursor film causes a significant increase in dissipation.

As shown in Fig. 5, the effective interface potential 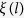 is related to the equilibrium contact angle, *θ*_*e*_, as


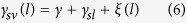



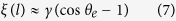


A high negative value of 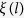 or, subsequently, the repulsive disjoining pressure is related with the limited spreading of the precursor film^12^,^40^. The spreading of a precursor film could be observed by freezing the liquid droplets and monitoring them using a cryogenic scanning electron microscope (Cryo-SEM). As shown in Fig. 5, a large precursor film was observed for the water droplet on the Si (hydrophilic) substrate, but in the case of PTFE (hydrophobic), the length was relatively short. Considering the adiabatic case, the length of the precursor film is proportional to the square-root of the initial spreading coefficient 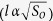
7. The initial spreading coefficient 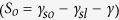 is >0 for the hydrophilic surfaces, whereas it is ≤0 for the hydrophobic surface. Thus, for the hydrophilic surfaces, the length of the precursor film is much longer compared to the hydrophobic surfaces (Fig. 5). This result was further supported by a previous study by Heslot^41^.

The length of a precursor film, which is controlled by intermolecular forces, depends on the combined effect of (i) the van der Waals, (ii) electrostatic and (iii) structural (solvation and hydration) forces.

First, the van der Waals attraction force for the sphere-plane case is given as^40^


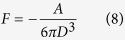


where *D* is the distance between the surfaces, and *A* is the Hamaker constant. The dispersion component of the van der Waals force for two macroscopic phases 1 and 2 interacting across medium 3 is estimated using the Lifshitz theory^40^


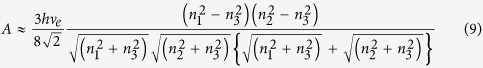


which relates the Hamaker constant, *A*, with the refractive indices, *n*, the Boltzmann’s constant, *k*, the absolute temperature, *T*, and the absorption frequency, *ν*_*e*_. Using the value given in supplementary Table S2, the value of the Hamaker constant for the Si/water system is calculated as *A*_*Si*_ ≈ 14.26 × 10^−20^ and for the PTFE/water system as *A*_*PTFE*_ ≈ 3.68 × 10^−20^. Thus, for the Si/water system, the van der Waals attraction force is approximately 4 times higher than that for the PTFE/water system.

Second, the electrostatic component of the disjoining pressure arises due to the presence of H^+^and OH^-^ ions in water. These ions result in the electrical double layer (EDL)^40^ at the Si/SiO_2_ surface and subsequently strongly interact with the hydroxyl group, resulting in an increase in the concentration of these ions near the solid surface. By contrast, a weak interaction exists between the fluorine component of the PTFE and hydrogen ions. Thus, EDLs with opposite charges are formed in both cases. Consequently, a strong attractive force between the EDLs of the Si/SiO_2_ and water-air interface presents an attractive force, whereas the EDL of the PTFE and water-air interface present a repulsive force.

Third, the structural forces are also repulsive for the hydrophobic surfaces due to strong hydration forces^40^. In general, a structural component of the disjoining pressure is repulsive for the interacting hydrophobic surface and water because of strong hydrophobic and hydrophilic attraction forces, whereas it is attractive for hydrophilic surfaces and water.

Overall, the three intermolecular forces present an attractive disjoining pressure between water droplets and hydrophilic surfaces, and the precursor film is spontaneously formed. By contrast, on hydrophobic surfaces, the three forces give rise to a repulsive interaction, retarding the formation of a precursor film.

From the description above, it becomes evident that the dominance of a precursor film limits the relative contribution of other factors in dissipation. Popescu *et al.*^7^ also indicated the importance of large spreading power on hydrophilic surfaces, ultimately leading to the failure of hydrodynamic analysis. This is the reason why the change of viscosity presents minor effects on the apparent contact angle of the hydrophilic surface. By contrast, the other two dissipation mechanisms (viscous dissipation and line friction) must be considered in semi-hydrophobic and hydrophobic cases because of the limited formation of the precursor film on hydrophobic (PTFE) and semi-hydrophobic (DLC) surfaces.

### Viscous wetting model

As shown in the previous section, viscous forces play an important role in the wetting of hydrophobic and semi-hydrophobic surfaces, especially textured surfaces. This force influences the droplet profile and therefore the advancing macroscopic (apparent) contact angle. We developed a model to predict the apparent contact angle for textured surfaces by considering the effect of the viscous force. We followed the mechanistic approach used by de Ruijter *et al.*^42^ and de Gennes^3^, in which the balance between the driving capillary forces and the resisting viscous forces controls the apparent contact angle of the droplet. de Ruijter *et al.*^42^ formulated a time-based close-form equation from the balance of the driving and dissipative forces for determining the contact angle on the flat surfaces. We extended this approach to the textured surfaces.

The driving capillary force per unit length for the textured surface with roughness factor 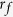 obtained from the potential energy of the solid-liquid-gas system is equal to


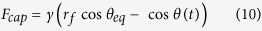


This is the modified form of the expression used by de Ruijter *et al.*^42^, where *θ*_*eq*_ is the Young’s contact angle. Equation (9) is similar to the Wenzel model and takes into consideration the effect of texture. In the case of a textured surface, the total viscous force is given as


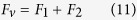


where *F*_*1*_ is the viscous force from the bottom flat surface and *F*_*2*_ is the viscous force of the textured geometry. From the hydrodynamic theory, the viscous force from the bottom flat surface per unit length of the contact line scales to^36^


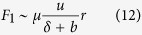


where *δ* is the boundary layer thickness controlled by the shear strain rate. Both velocity magnitude, *u*, and slip length, *b,* determine the shear strain rates. Landav and Levich^38^ showed that *δ* scales as 0.94κ*Ca*^2/3^. Here, κ is the capillary length. In the case of Stokes’ flow, the viscous resistance obscured by the cylindrical geometry was found to be^36^,^43^


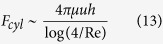


where *Re* is the Reynolds’s number, and *h* is the height of the pillar. For periodically arranged cylindrical pillars with a periodicity *p* (pitch), the viscous force transpassing a single pillar geometry scales to^43^


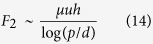


where *d* is the diameter of the pillar. With *r/p*^*2*^ pillars per unit width of the contact line, the total viscous force per unit length scales as


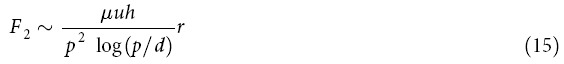


By balancing the driving and resisting forces, we obtain the following equation


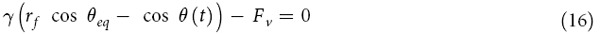


The value of *θ(t)* at which both the forces balance each other gives the apparent contact angle. The apparent contact angle is a function of the geometric parameters of the textured surface, the spreading velocity, and the unknown contact radius, *r*. With the spherical cap assumption, the basic trigonometry relates the contact angle, initial drop radius *R*_*o*_ and the contact radius as


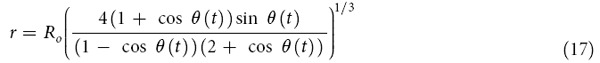


Furthermore, spreading velocity is a function of a droplet radius, time and fluid properties; it has a power-law relation with time^33^.


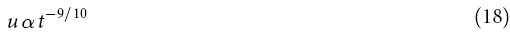


This leads to the following relation between velocity and time with *a* the fitting parameter obtained from best fit curve to the experimental results


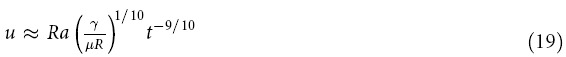


Substituting the respective values from equations (3), (12), (16) and (18) into equation (14) gives


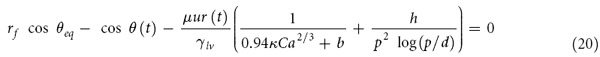


**Figure 6:**
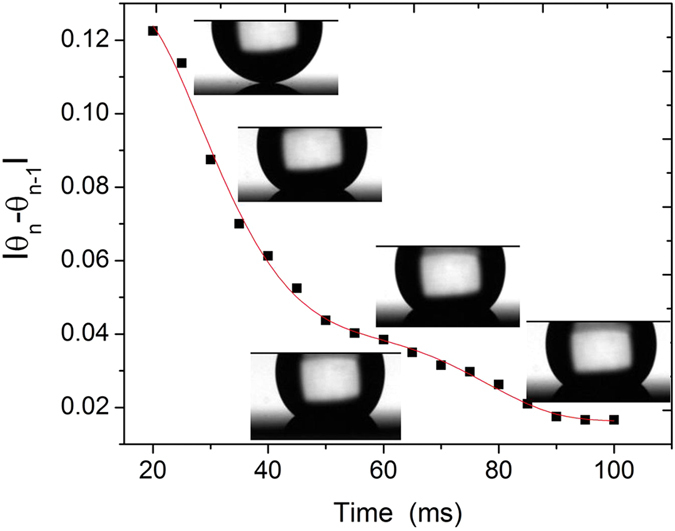
An example of the converged time iteration curve from our model used for estimating the value of the apparent contact angle, *θ*(*t*). The difference 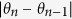 was plotted vs time *t* and the value of *θ*(*t*) was obtained after the solution converged. A large difference in the value of *θ*(*t*) indicates non-equilibrium state. The water droplet images shown in the inset demonstrate the droplet shape at different time instances and complements our approach.

This non-linear expression has two unknown variables, *θ* and *t*. We solved this expression numerically using the principle of minimisation and the Secant method. In the first step, for a given time step, *t*, *θ* was determined corresponding to the force-balance of the driving and resisting forces. For this, *θ* was varied from 0° to 180° in steps of 0.1°. The aim is to find the *θ* at which the driving and resisting forces balance each other or minimises the difference between them. In the second step, the time was advanced by 1 ms, and the first step was repeated. This process continued until the solution converges (Fig. 6). From the observed cross-over time (10–20 ms) from inertia to the viscous force-dominant regime in Fig. 2, the starting value of the time iteration was set equal to 20 ms, similar to the transition point^44^.


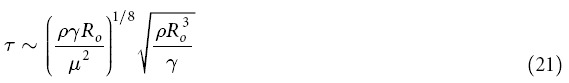


As shown in Fig. 6, the change of the contact angle in each time step, |*θ*_*n*_ − *θ*_*n*−*1*_|, decreases as time elapse, and reaches nearly constant value of ~0.02 at the time point of 100 ms. Because the |*θ*_*n*_ − *θ*_*n*−*1*_| value is not zero at this time point, the contact line still advances, but the difference remains almost constant.

**Figure 7:**
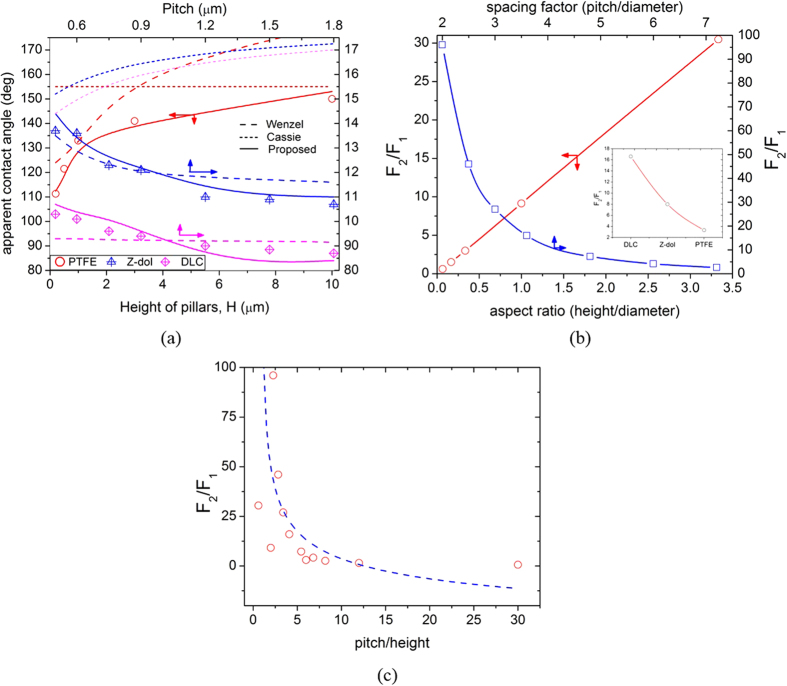
(**a**) Comparison between the results of macroscopic apparent contact angle predicted by our model (solid line) and the Wenzel model (dotted line), with the experimental results (symbols) for the micro and nano textured DLC, Z-dol and PTFE surfaces with different heights and pitch. Detailed dimensions of the texture geometry are given in Table S3. (**b**,**c**) Relationship between the ratio of viscous forces of the textured (F_2_) to the flat (F_1_) surface, and the geometric parameters. Inset shows the effect of surface chemistry (slip length) on F_2_/F_1_ for the same roughness factor.

The model was validated by comparing the calculated estimations with the experimental contact angle of water droplets on the semi-hydrophobic and hydrophobic textured surfaces. For comparison, micro and nanoscale pillars of different height and pitch were used (Supplementary Table S3). The wetting states of the patterned surfaces were estimated using a comprehensive Gibbs free energy simulation model^45^ and the finite element method^46^. Among the batch of the patterns, only the patterns exhibiting the Wenzel state were used. The experimental contact angles were compared with the calculated values from this model as well as those obtained from the variants of the Wenzel and Cassie-Baxter models^47^. In these variants of the Wenzel and Cassie-Baxter models, advancing angle was used in place of the equilibrium angle^47^. Figure 7(a) shows a significant difference exists in the trends of the experimental results and those of the modified Cassie-Baxter model (dotted line). As shown in Fig. 7(a), the proposed model (solid lines) showed values closer to the experimental results (symbols) compared with those from the modified Wenzel model (dashed lines). Such difference mainly originated from the consideration of hydrodynamic forces, which are not considered in the Wenzel model. This result indicated that viscous force plays an important role in the determination of apparent contact angle.

Furthermore, the influence of the geometric parameters of the patterns (aspect ratio and spacing factor) on the ratio of the viscous force for the textured to the flat surfaces is shown in Fig. 7(b) and (c). The ratio of viscous forces for the textured surface has a direct relationship with the aspect ratio (ratio of height to diameter of a pillar), which is due to the increase in the projected area of the pillar. However, the ratio of viscous forces showed an inverse logarithmic relation with the spacing factor (ratio of pitch to diameter of a pillar). The ratio of viscous forces increases rapidly for a spacing factor ≤3, whereas, for a spacing factor >7, the viscous forces of the texture are comparable to those of a flat surface. This difference in ratio of viscous forces is due to the decrease of shear strain rates with the increase in spacing between the pillars.

The inset in Fig. 7(b) shows a relationship between the surface chemistry and the ratio of the viscous forces (*F*_*2*_/*F*_*1*_). When considering DLC, Z-dol, and PTFE, the highest ratio of viscous forces was obtained on DLC-coated surfaces, whereas the lowest ratio was found in PTFE-coated surfaces.

This behaviour is related to the slip length of the surface. Among all surfaces investigated, DLC has the smallest slip length, which resulted in highest strain rates, whereas the PTFE demonstrated the lowest strain rates as it has the largest slip length. Furthermore, we plotted the variation of the viscous forces with respect to the ratio of pitch to height of a pillar (Fig. 7c). The results in Fig. 7(b,c) indicate that the influence of pitch (spacing) on the viscous force becomes dominant for *p* ≤ 5*h* and/or *p* ≤ 3*d*. Above this range, the viscous forces are mainly controlled by the height.

### Summary

In this study, a theoretical model to more accurately predict the apparent contact angle of a liquid drop was proposed by considering the role of dissipative processes, particularly viscous forces. For this purpose, we measured the apparent contact angle of liquid drops with various viscosities and demonstrated that the effect of viscous forces is more important in textured surfaces with semi-hydrophobic and hydrophobic surface chemistry. Based on these observations, we formulated a model that can predict the apparent contact angle of a liquid drop on arbitrary textured surfaces by equating the driving capillary force and resisting viscous force. This model can predict contact angle much more accurately than the conventional Wenzel model, and the correspondence between the experimental value and theoretical estimation further supports the importance of including viscous forces in the determination of contact angle of textured hydrophobic surface. It is believed that this model can be applied in various engineering applications that require an estimation of the wetting properties of surfaces.

## Methods

### Preparation of a textured surface with varying surface chemistry

Textured Si surfaces consisting of cylindrical micro- and nanoscale pillars were fabricated from conventional MEMS processes such as photolithography and deep reactive ion etching (deep RIE). Two main types of patterns were fabricated to investigate the effect of height *H* and pitch *P*. For micro-textures, cylinder micro-pillars arranged in a square lattice with a diameter of *D* = 3 μm; *P* = 6 μm and different pillar heights, *H* (0.2, 0.5, 1, 3, 10, 20, and 25 μm; for further information supplementary Table S3) was prepared. For nano-textures, cylindrical nanopillars arranged in square lattice with *D* = 220 nm; *H* = 250 nm and different *P* (500, 600, 750, 90, 1200, 1500 and 1800 nm; for further information please see supplementary Table S2). Results showing the effect of *P* on contact angle were reported earlier elsewhere^48^. After the surface patterning, the micro- and nanopatterns were subsequently coated with a 20-nm thick layer of polytetrafluoroethylene (PTFE), perfluoropolyether (Z-Dol) and diamond-like carbon (DLC) to obtain surfaces with different wettabilites.

### Measurement of contact angles

The initial droplet spreading experiments were carried out using high-speed imaging technique (Photron, IDP Express R2000) operating at 5,000 frames per seconds with a shutter speed of 0.0001 s. To study the effect of viscosity, liquids were prepared by mixing different proportions of glycerol (Sigma-Aldrich) and de-ionised (DI) water (Supplementary Table S1). Water and glycerol were selected due to their almost similar densities, surface tension, and high affinity for each other. The viscosity of these mixtures was measured using a Viscometer (Cannon Fenke) at a temperature of 24 ± 0.5 °C and 45% relative humidity with an accuracy of ±5%. Density of these mixtures was determined by weighing a known volume using micro-balance, and surface tension was calculated using the sessile drop method with the help of Goniometer. The apparent contact angles were measured with the sessile droplet method. Reported values are the constant values corresponding to the quasi-static condition. For each case, experiments were repeated five times and average results are reported. The value of the contact angle was determined with an accuracy of ±1°.

## Additional Information

**How to cite this article**: Grewal, H. S. *et al.* Role of Viscous Dissipative Processes on the Wetting of Textured Surfaces. *Sci. Rep.* **5**, 14159; doi: 10.1038/srep14159 (2015).

## Supplementary Material

Supplementary Information

Supplementary Movie 1

Supplementary Movie 2

## Figures and Tables

**Figure 1:**
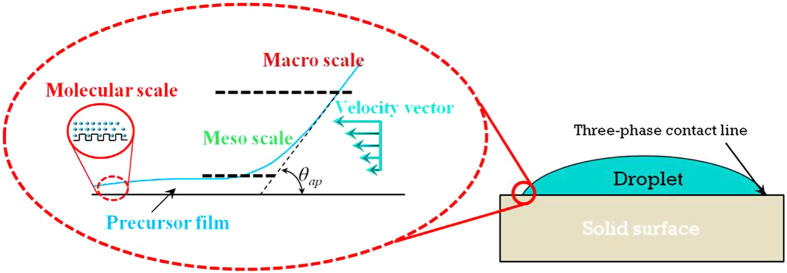
The apparent contact angle is a multiscalar property. At each scale, different forces dominate and influence the contact angle^3^,^4^.
