# Supplementary material for: Role of Viscous Dissipative Processes on the Wetting of Textured Surfaces
Source: Sci Rep. 2015 Sep 22;5:14159. doi: 10.1038/srep14159 (PMC4585758; doi:10.1038/srep14159)
Supplement: Supplementary Information [file srep14159-s1.pdf]

SUPPLEMENTARY INFORMATION

Role of Viscous Dissipative Processes on the Wetting of Textured Surfaces

H. S. Grewal, Hong Nam Kim, Il-Joo Cho, Eui-Sung Yoon*

Center for Biomicrosystems, Korea Institute of Science and Technology (KIST), Seoul, Republic of Korea

*Correspondence: E.-S. Yoon, Ph.D.
e-mail: esyoon@kist.re.kr
Phone: +82-2-958-5651
Fax: +82-2-958-6910

Supplementary Table S1: Physical properties of glycerol and water mixture used in wetting studies

Glycerol (%)/Water (%)	Viscosity (mPa S)	Surface tension (mN/m)	Density (Kg/m^3)
100/0	1268.2	63.0	1270.1
95/5	782.3	63.3	1253.0
90/10	263.5	63.7	1242.5
85/15	123.4	64.6	1231.4
75/25	41.4	65.1	1208.5
50/50	6.7	68.0	1146.0
0/100	0.98	71.2	998.0

Supplementary Table S2: Value of the parameters used in Eq (8) for calculating the Hamaker constant

Material	Dielectric constant, ε	Refractive index, n	Absorption frequency, ν_e
Water	80	1.334	3×10^{15}
PTFE	2.1	1.359	2.9×10^{15}
Si	11.68	3.48	3×10^{15}

Supplementary Table S3: Dimensions of the micro and nano cylindrical patterns used for wetting studies

Case no.	Dimensions of micro-pillar									Surface material
I	Diameter *D* (μm)	Pitch *P* (μm)	Height *H* (μm)							PTFE
	3	6	0.2	0.5	1	3	10	20	25	
II	Diameter *D* (nm)	Height *H* (nm)	Pitch *P* (nm)							Z-Dol and DLC
	250	220	500	600	750	900	1200	1500	1800	
